# Nurse-Led, Remote Optimisation of Guideline-Directed Medical Therapy in Patients with Heart Failure and Reduced Ejection Fraction Across Australia

**DOI:** 10.3390/jcm14155371

**Published:** 2025-07-30

**Authors:** Gabrielle Freedman, Racheal Watt, Enayet Karim Chowdhury, Kate Quinlan, David Eccleston, Andrea Driscoll, James Theuerle, Leighton Kearney

**Affiliations:** 1 Department of Cardiology, Austin Health, Heidelberg, VIC 3084, Australia; andrea.driscoll@deakin.edu.au (A.D.); james.theuerle@austin.org.au (J.T.); 2 Advara HeartCare, East Melbourne, Melbourne, VIC 3002, Australia; racheal.watt@advaraheartcare.com (R.W.); enayet.chowdhury@advaraheartcare.com (E.K.C.); kate.quinlan@advaraheartcare.com (K.Q.); david.eccleston@advaraheartcare.com (D.E.); 3 School of Public Health, Monash University, Melbourne, VIC 3004, Australia; 4 Faculty of Medicine, Dentistry and Health Sciences, University of Melbourne, Melbourne, VIC 3010, Australia; 5 Monash Health, Clayton, VIC 3800, Australia; 6 Centre for Quality and Patient Safety Research—Monash Health Partnership, Deakin University, Geelong, VIC 3220, Australia

**Keywords:** heart failure with reduced ejection fraction, guideline directed medical therapy, left ventricular ejection fraction, nurse, remote, titration, artificial intelligence, natural language processing

## Abstract

**Background/Objectives**: Guidelines recommend patients with heart failure with reduced ejection fraction (HFrEF) receive four-pillar heart failure (4P-HF) therapy, which significantly reduces cardiac morbidity and mortality. However, implementing these guidelines effectively into clinical practice remains challenging. **Methods**: Patients with HFrEF on submaximal 4P-HF therapy were identified from a large, multicentre Cardiology network database using a natural language processing tool, supported by manual file review. A nurse-led, remotely delivered, medication uptitration program aimed to optimise therapy in this real-world cohort. **Results**: The final cohort included 2004 patients with a mean age of 72.7 ± 11.6 years. Utilisation of 4P-HF increased from 11.1% at baseline to 49.8% post intervention, and each individual medication class increased significantly post intervention (all *p* < 0.001). The largest increase was observed with the use of sodium–glucose cotransporter 2 inhibitors, which rose from 17.3% to 73.9%, followed by mineralocorticoid receptor antagonists (51.6% to 65.7%), beta-blockers (88.4% to 97.0%), and angiotensin-converting enzyme inhibitors/angiotensin receptor blockers/angiotensin receptor blocker–neprilysin inhibitors (89.8% to 96.4%). In patients on submaximal therapy, barriers were documented in all cases. Following medication optimisation, left ventricular ejection function (LVEF) improved significantly (38.5% ± 10.8% vs. 42.5% ± 11.7, *p* < 0.001). **Conclusions**: This nurse-led, remotely delivered, medication optimisation program significantly improved the adoption of 4P-HF therapy and LVEF in patients with HFrEF. The program demonstrates a practical, scalable solution for the optimisation of HFrEF therapy across a large healthcare network.

## 1. Introduction

Heart failure (HF) is a chronic disease associated with substantial disability, impaired quality of life, and a sobering four-year mortality of 40% [1]. HF results in a major expense to healthcare systems that are disproportionately weighted towards costly, hospital-based care [2]. In Australia, this equates to a total cost of AUD 2.7 billion dollars annually, which is expected to increase to AUD 3.8 billion by 2030 [3].

A large body of evidence has informed current guidelines recommending four-pillar heart failure (4P-HF) therapy, which when implemented leads to a substantial reduction in hospitalisation, improved quality of life, and improved survival [4,5,6]. Compared with no treatment, 4P-HF therapy in heart failure with reduced ejection fraction (HFrEF) patients improves life expectancy by up to 5 years [4].

Following the 2021 European Society of Cardiology Heart Failure guidelines, the Cardiac Society of Australia and New Zealand issued a 2022 consensus statement endorsing early initiation of all four first-line pharmacotherapies, moving away from a stepwise approach [6,7]. Furthermore, recent data from the STRONG-HF trial demonstrated the safety of the early escalation of therapy in reducing the risk of death and readmission for HF, and this is increasingly influencing medication sequencing practices [8,9,10].

However, translating recommendations into clinical practice has proven to be a formidable challenge [11]. For example, in a US cohort of 3518 HFrEF patients, only 22.1% of patients who were eligible for guideline-directed medical therapy (GDMT) were prescribed it, and only 1.1% of patients achieved maximal doses [12]. More recent studies examining the uptake of sodium–glucose cotransporter 2 inhibitors (SGLT2i) continue to highlight suboptimal implementation of comprehensive four-pillar therapy, and historically, target doses are uncommonly achieved in GDMT [13,14,15,16,17].

**Figure 1:**
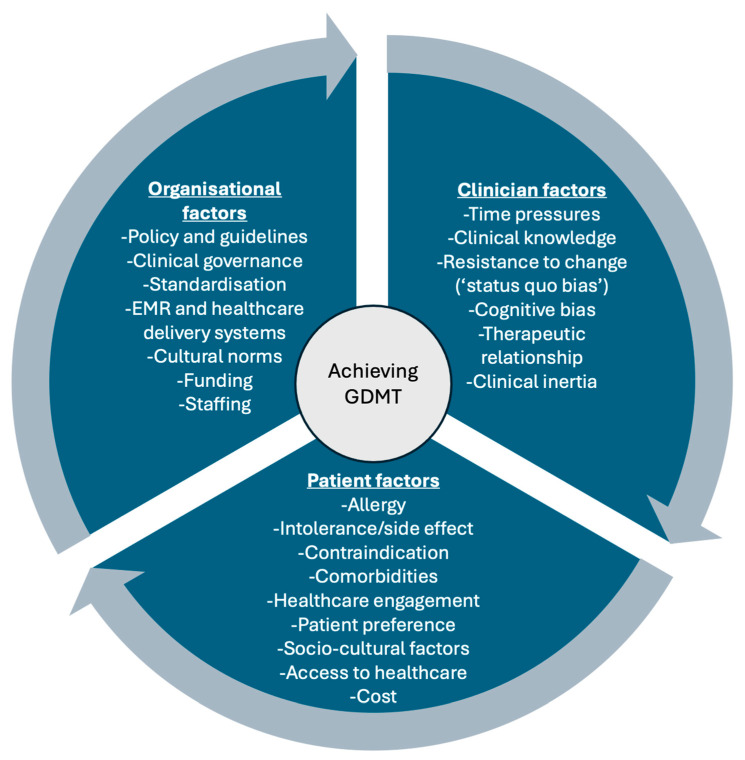
Interacting factors that influence the uptake of guideline-directed medical therapy, including organisational factors, clinician factors, and patient factors. EMR, electronic medical record; GDMT, guideline-directed medical therapy.

Multiple factors contribute to this prescribing gap (Figure 1). While patient-level issues, such as hypotension and renal impairment, are common, clinician and organisational factors also play a significant role [18]. ‘Clinical inertia’ is increasingly recognised in heart failure management and refers to a clinician’s failure to escalate evidence-based therapy in the absence of clear contraindication [19,20,21]. Contributing factors include guideline misconceptions, limited confidence with newer therapies, time constraints, and system-level issues, such as workflow design [22,23,24]. These overlapping barriers can result in under-prescribing, often with the inadequate documentation of rationale for omission [11,15].

Community-based HF management programs focused on the implementation of GDMT have been shown to improve clinical outcomes and reduce hospital utilisation [25,26]. A recent meta-analysis found that patients attending nurse-led titration clinics were 97% more likely to be optimised on GDMT compared with patients who did not, supporting the class 1a recommendation for HF nurse involvement in clinical guidelines [6,27,28].

However, given the scope of the management gap, population-level programs are needed. Remote healthcare has gained popularity in recent years, overcoming geographical barriers while maintaining high-quality care [24,29]. Additionally, artificial intelligence (AI) technologies, such as natural language processing models (NLPs) can efficiently interrogate and extract data contained within large electronic medical record (EMR) datasets [30]. In this way, NLPs can be used to identify sub-optimally managed HFrEF patients appropriate for intervention, leveraging technological advancements that support scalable interventions in HF management.

The aim of this project was to implement a nurse-led, remotely delivered, medication optimisation program, supporting 2000 patients with HFrEF to achieve maximally tolerated 4P-HF therapy over a 12-month period.

## 2. Materials and Methods

### 2.1. Patient Population

Patients with HFrEF were recruited from Advara HeartCare, a large, multicentre, private cardiology network with 62 cardiology practices across metropolitan and regional locations in Australia. In Australia, outpatient cardiac care is subsidised through Medicare, with access to public hospitals and cardiology clinics at no or minimal cost to patients [31]. Private providers, such as Advara HeartCare, operate independently of the public system and usually involve out-of-pocket costs.

HFrEF was defined according to the universal definition and classification of heart failure [28]. Patients were included if they were not on maximal doses of 4P-HF therapy and they had a previous LVEF ≤ 40% within 2 years from enrolment, irrespective of their most recent LVEF. Patients were excluded if they were over 85 years, had a competing comorbidity with an expected survival <12 months, or were deemed medically frail by the treating cardiologist. Final approval for program participation was at the discretion of the managing cardiologist. Allergy or contraindication to therapy, chronic kidney disease stages 4–5, hyperkalaemia, and hypotension were not exclusion criteria. The aim of this initiative was to up-titrate a real-world population onto their maximally tolerated dose of GDMT.

Four-pillar HF therapy consisted of an angiotensin-converting enzyme inhibitor (ACEI), angiotensin receptor blocker (ARB), or angiotensin receptor blocker–neprilysin inhibitor (ARNI); beta-blocker (BB); mineralocorticoid receptor antagonist (MRA); and SGLT2i [7,32]. During the program period, local prescribing guidelines for SGLT2i required symptomatic HF with a LVEF ≤ 40%. ARNI prescription required symptomatic HF with LVEF ≤ 40% after 3–6 months of maximal tolerated ACEI/ARB [33]. Maximal drug doses are defined in the Supplementary Material (Table S1) and were based on guidelines at the time of the study [34].

### 2.2. Patient Recruitment and Natural Language Processing

Potentially suitable HFrEF patients who might benefit from HF medication review were identified via the following three main sources: (1) NLP tool interrogation of the Advara HeartCare EMR (HealthTrack Medical Systems, Cascade 430, and Dolomites449); (2) periodic EMR data extracts with search parameters including LVEF, HFrEF diagnosis, HFrEF specific medications; and (3) direct referral by cardiologists to the program. This information was collated with other clinically relevant structured data fields within the EMR, such as age, echocardiography parameters, pathology results, and administrative details, such as next consult date and treating cardiologist, to assist with efficiency of follow-up for nurse involvement.

The NLP tool was designed in conjunction with Max Kelsen Pty. Ltd., Spring Hill, Australia. In March 2021, the model interrogated electronic medical records of 396,278 patients who were seen within the Advara HeartCare network from 1 January 2015 to 28 February 2021, inclusive. The search identified 22,053 patients with HFrEF. The NLP model integrated unstructured clinical letters and investigation results with structured data, such as echocardiography parameters and laboratory biomarkers, to create a deterministic, rule-based NLP pipeline. This pipeline extracted key entities and corresponding values, applying expert-defined thresholds to identify the presence of HFrEF. A comprehensive description of the NLP methodology is provided in the Supplementary Materials.

From this large patient cohort, patients were selected for HF nurse manual file review based on having undergone a cardiologist review within the preceding 24 months and with a scheduled cardiologist review within the upcoming 12 months. The nurse reviewed 3483 patient files to determine suitability for inclusion in the program based on current demographic, clinical, and medication data. Where required, pharmacists provided updated medication lists. Separate patient consent was not required, as this initiative was deemed to be a quality assurance activity conducted within the standard of care framework at Advara HeartCare, for which patient consent had been previously obtained.

### 2.3. Nurse-Led Titration Program

The program was conducted over 15 months from February 2023 to May 2024, which was extended from 12 months to support effective implementation. It was delivered by a single experienced cardiac nurse in collaboration with treating cardiologists. The nurse reviewed patient records to identify medication optimisation opportunities and signalled these to the cardiologist prior to the patient’s usual consultations, and medication changes were enacted by the cardiologist if deemed appropriate. The nurse did not attend appointments; however, they were responsible for remotely supporting the implementation of medication changes. This included assisting with script management, monitoring for patient symptoms and side effects, coordinating investigations, and providing patient education. Clinically relevant information was escalated to the cardiologist. Nurse–patient communication occurred via telephone, email, or text. Once the cardiologist determined the patient’s therapy was optimised, they were discharged from the program.

### 2.4. Program Coordination

Cardiologists were engaged through state-based educational events led by local State Leads, which provided updates on HFrEF guidelines, the role of nurse-led care, and the program’s structure. A dedicated project manager coordinated the education events, facilitated stakeholder engagement, and assisted the HF nurse with data management.

### 2.5. Statistical Analysis

Categorical variables are presented as frequencies and percentages, while continuous variables are expressed as either mean ± standard deviation (SD) or median (inter quartile range, IQR), depending upon the data distribution, as determined by Skewness–Kurtosis testing. McNemar’s test and paired *t*-tests were used to assess differences in categorical and continuous variables, respectively, between baseline and study completion. The analysis was conducted using Stata version 19.5, with no imputation of missing data.

## 3. Results

### 3.1. Demographic Data

**Figure 2:**
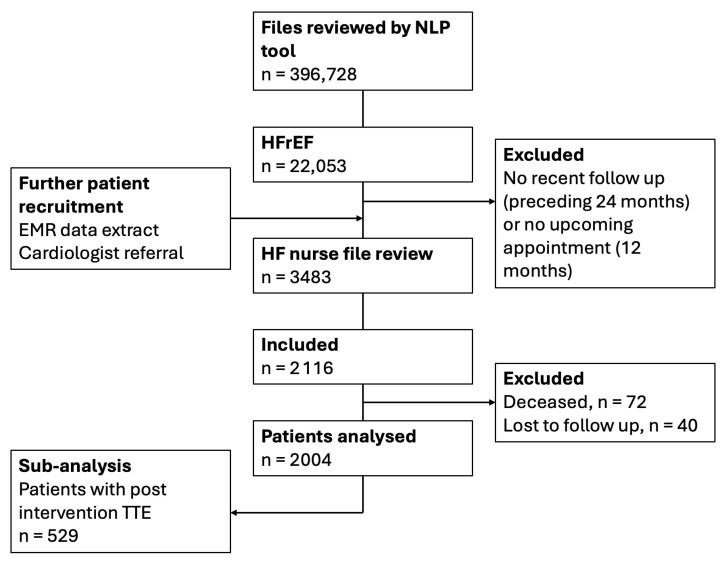
Study flow chart outlining patient recruitment and number of patients analysed. n = number of patients. EMR, electronic medical record; HF, heart failure; HFrEF, heart failure with reduced ejection fraction; NLP, natural language processing, TTE, transthoracic echocardiogram.

A total of 2116 patients were included, and of those, 2004 completed the program. The results of this report are based on the completed patient cohort (Figure 2). The mean patient age was 72.7 ± 11.6 years, and 72.1% were male (Table 1).

### 3.2. Four-Pillar Therapy Uptake

**Figure 3:**
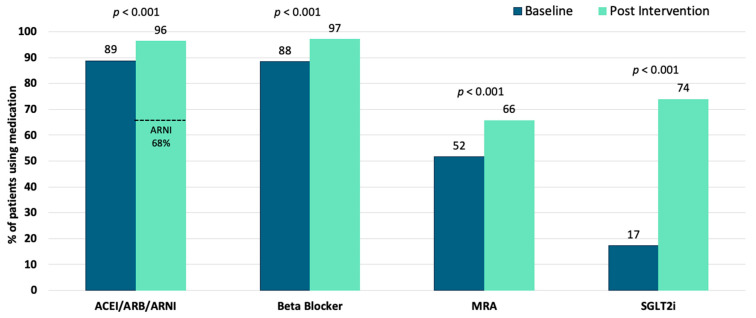
Medication class uptake at program baseline and following the nurse-led intervention. Bar graphs represent the proportion of the cohort using a class of medication. *p* values demonstrate significant increases in all four medication classes. ACEI/ARB/ARNI; angiotensin converting enzyme inhibitors, angiotensin receptor blockers, angiotensin receptor blocker–neprilysin inhibitors; MRA; mineralocorticoid receptor antagonists; SGLT2i, sodium–glucose cotransporter 2 inhibitors.

At baseline, 11.1% (222/2004) of patients were on 4P-HF therapy at submaximal doses, and at study completion, 4P-HF therapy increased significantly to 49.8% (998/2004); *p* < 0.001. Similarly, the combined proportion of patients on three- or four-pillar therapy increased substantially during the study period, from 50.7% (1016/2004) to 84.8% (1700/2004); *p* < 0.001. The uptake of all four medication classes increased significantly during the program, with SGLT2i seeing the greatest increase from 17% (1481/2004) (Table 1 and Figure 3). MRA uptake increased from 51.6% (1035/2004) to 65.7% (1316/2004), *p* < 0.001; BB uptake increased from 88.4% (1771/2004) to 97.0% (1944/2004), *p* < 0.001; and ACEI/ARB/ARNI uptake increased from 88.8% (1779/2004) to 96.4% (1932/2004), *p* < 0.001. At study completion, ARNI were prescribed in 67.8% (1359/2004) of patients. Patients who achieved 4P-HF therapy during the program were younger (71.3 ± 11.3 years vs. 74.1 ± 11.8 years; *p* < 0.001). A greater proportion of patients living in regional Australian communities achieved 4P-HF therapy compared with patients living in metropolitan areas (52.9%, 387/732, vs. 48.1%, 611/1271, *p* = 0.04). There was no difference in rates of 4P-HF therapy based on gender (*p* = 0.78).

### 3.3. Barriers to Four-Pillar Therapy Uptake

**Figure 4:**
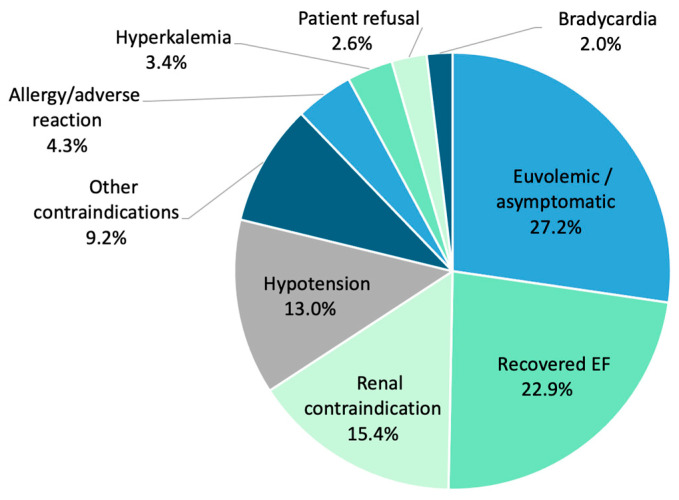
Clinical barriers preventing escalation of four-pillar heart failure therapy to maximal doses, as demonstrated by percentage. EF, ejection fraction.

A total of 50.2% (1006/2004) of participants were unable to achieve 4P-HF therapy. All patients receiving less than 4P-HF therapy had a documented reason for medication non-uptake. The most cited reasons included euvolemic/asymptomatic status (27.2%, n = 274), recovered LVEF (22.9%, n = 230), renal contraindication (15.4%, n = 155), hypotension (13.0%, n = 131), other contraindications (9.2%, n = 93), allergy/adverse reaction (4.3%, n = 43), hyperkalaemia (3.4%, n = 34), patient refusal (2.6%, n = 26), and bradycardia (2.0%, n = 20) (Figure 4). An eGFR < 30 mL/min/1.73 m^2^ was not significantly associated with the omission of 4P-HF therapy (*p* = 0.13).

### 3.4. Association Between Left Ventricular Ejection Fraction and Four-Pillar Therapy

The baseline LVEF for the entire cohort of 2004 patients was 38.3% ± 10.8%. The SD reflects the inclusion of patients with LVEF > 40% due to the time lag between NLP-based data extraction in March 2021 and study commencement in February 2023. At the time of program commencement, 657 patients (32.8%) demonstrated improvements in LVEF to >40%; however, these participants were retained in the cohort given their prior diagnosis of HFrEF. Of the 1347 patients with persisting LVEF ≤ 40%, the mean LVEF was 32.5 ± 6.6%.

LVEF was not routinely re-assessed as part of the program; however, 529 patients (26%) underwent clinically indicated echocardiograms after the intervention was completed. Within this cohort, there was a significant increase in mean LVEF from 38.5 ± 10.4% at baseline to 42.5 ± 11.7% post maximal titration (*p* < 0.001). Patients observed significant improvements in their ejection fraction, regardless of whether they reached 4P-HF therapy or not.

### 3.5. Program Delivery Resources

The program was delivered by one full-time registered HF nurse who supported the medication up-titration of 2004 patients across Australia. Over the 15-month period this translated into 1.2 nurse-hours per patient optimised. The median number of cardiology consultations per patient was 2 (IQR 1-4). Nurse–cardiologist interactions were not routinely recorded. A project manager helped coordinate the program and dedicated 1 day per week over the study period. A lead cardiologist oversaw the national program.

## 4. Discussion

This study showed that a remote, nurse-led program, assisted by AI technology, can effectively enhance the adoption of 4P-HF therapy in patients with HFrEF. We observed a 38.7% increase in 4P-HF therapy uptake over the study duration (11.1% at baseline to 49.8% post intervention, *p* < 0.001). Specifically, use of SGLT2i rose by 56.2% (17.3% to 73.9%, *p* < 0.001), demonstrating that targeted interventions can help enhance the adoption of new therapies [35,36,37,38]. MRAs (51.6% to 65.7%), BB (88.4% to 97.0%), and ACE/ARB/ARNI (89.8% to 96.4%) all increased significantly (all *p* < 0.001). In addition, we observed a 4% improvement in LVEF following medication escalation in a subgroup of patients with follow-up echocardiography.

All subjects on submaximal 4P-HF therapy had a documented reasons for non-uptake, with 50.2% of participants unable to achieve full doses of 4P-HF therapy. For this group, the program systematically captured barriers to medication optimisation, offering valuable insights into the practical limitations of heart failure management. Contemporary GDMT targets are based on randomised control trials (RCTs) often involving younger, healthier, and less diverse populations; however, this can limit their generalisability to routine clinical settings [22]. Furthermore, the significant GDMT medication gap has prompted questions around whether full-dose 4P-HF therapy is feasible, and necessary, for all patients [37,39]. In contrast, this program applied non-restrictive inclusion criteria (for example including 95 patients with chronic kidney disease stage 4–5), reflecting a more comorbid and heterogenous cohort. As such, the medication uptake rates may represent more realistic, achievable targets in everyday practice.

While temporal trends and increased familiarity with new guidelines are likely to increase medication uptake, comparative data support the likelihood that the current results may, at least in-part, be intervention-driven [40]. Persistently low uptake of 4P-HF therapy has been reported across several contemporary studies; for instance, only 37.2% of patients in the TITRATE-HF registry (Netherlands, 2022–2024) received all four guideline-recommended therapies, with 15.3% in a large North American cohort of 33,000 patients by Greene et al. (2021–2023) and 37.6% in Turkish cohort by Kocabas et al. (2023) [16,17,41]. Within the current program, we were able to increase 4P-HF therapy from 11% to 50%, while simultaneously escalating therapy to maximally tolerated doses, reflecting the value of a directed outpatient HF therapy management program compared with usual care. This is particularly meaningful given that, in some cohorts, as few as 1% of patients achieve maximal doses of therapy, suggesting most are not titrated to their individual therapeutic targets [17].

Reported SGLT2i use in the same cohorts was variable and ranged from to 23.5% (Greene et al.) to 50.6% (Kocabas et al.) to 65.4% (TITRATE-HF) [16,17,41]. While increasing familiarity with SGLT2 inhibitors is likely to drive greater uptake over time, the current study demonstrated robust post-intervention uptake of SGLT2i and a markedly steep trajectory from 17.3% to 73.9% over 15 months (a monthly increase of 3.7%). In contrast, more gradual trends are observed in real-world data, including McGrane et al. (8% to 25% over 12 months; 1.4% per month) and Laborante et al. (10.3% to 31.9% over 20 months; 1.08% per month) [38,42]. The comparatively rapid adoption in the current study suggests a potential program-specific effect beyond background prescribing trends. Furthermore, evidence from longitudinal studies over the past 10 years have demonstrated temporal increases in prescribing SGLTis and MRAs but not in long-established therapies, such as BB or ACEI/ARB [43,44,45,46,47]. In contrast, our study reported significant increases in treatment with all four HF drug classes, supporting the value of a dedicated, nurse-led HF intervention in addressing therapeutic inertia [23,48].

By leveraging remote care and nursing expertise, the program delivered high-quality GDMT optimisation across a diverse, nationwide population. While the majority of patients enrolled in this study resided in metropolitan areas (67.3%), a greater proportion of regional patients achieved 4P-HF therapy compared to their metropolitan counterparts (52.9% vs. 48.1%; *p* = 0.04). This demonstrates the value of remote models of care in improving healthcare equity for geographically dispersed populations [49,50]. In Australia, multidisciplinary HF nursing programs are disproportionally located in metropolitan areas, creating a paradox whereby regional areas, which have higher rates of HF prevalence and age-standardised HF mortality, have less access to specialty HF care [51]. Similarly, a study found that digital consults incorporating data sharing and patient education via text and e-learning significantly increased GDMT use compared to usual care [50,52]. Our program extended this concept by integrating remote care together with a nurse-led model, harnessing the additive value of interdisciplinary support. Consequently, this initiative highlights the role of incorporating innovative models of care delivery in order to address longstanding disparities in healthcare access [53].

As a nurse-led program, nurses were placed at the forefront of patient engagement, education, and care coordination. While the average number of patient–cardiologist consultations was only two, nurses were able to support the up-titration of therapy across a large number of patients, with a relatively small amount of time investment (1.2 h per patient). This aligns with previous research demonstrating that HF nurse care facilitates higher and more rapid uptake of GDMT compared to usual care alone, which translates to fewer HF hospitalisations [25,48]. Given that 53.4% of cardiologists report workload as a major barrier to up-titrating GDMT, nurses offer a key opportunity to streamline workload and improve productivity [11,54].

In considering both barriers to GDMT and mechanisms to address them, this program incorporated several theoretical strengths. Firstly, its multifaceted approach (involving targeted education, clinical decision support, nurse reminders, and bidirectional multidisciplinary collaboration) helped counter therapeutic inertia, which is often driven by clinician education gaps, time constraints, and workload [20,23]. Challenges exist in modifying behaviour, saliently demonstrated by research on medication prompts whereby physicians receiving prompts were paradoxically less likely to escalate therapy due to ‘alert fatigue’ [55,56,57]. In contrast, this program’s success may have been influenced by the interpersonal communication between the nurse and the cardiologist within a collaborative team [55]. Additionally, at a patient level, nurse involvement has been shown to provide holistic psychosocial support, which can improve persistence and stability on therapy [25,58]. Systemically, the program also applied a uniform care approach, reducing variability in prescribing that may be influenced by cognitive bias, which has been linked to inequality in heart failure treatment delivery [59,60].

The integration of AI-assisted NLP tools was a key enabler in identifying patients on suboptimal therapy and targeting them for intervention at scale. While 2004 patients were included in this analysis, a total of 396,728 files were analysed by the NLP, demonstrating a level of reach that would be difficult to reproduce manually [30,61]. As such, international efforts are increasingly exploring how innovative digital strategies can be harnessed to improve heart failure management [24,29].

This study has several limitations that require acknowledgement. Most notably, the observational design and lack of a control arm limit causal inference, which would be better addressed through a randomised controlled trial (RCT) design. In addition, the effect size seen may have been influenced by temporal trends and enhanced prescriber familiarity with a new medication class, such as SGTL2is [43,44]. Another potential limitation is that the ‘Hawthorne effect’ may have played a role, a phenomenon whereby behaviour is modified simply as a function of being observed [62]. In this way, physicians may have been inclined towards greater intensification of GDMT than would be expected under non-study conditions. Double blinding of clinicians to the hypothesis could reduce this potential source of bias. The absence of medication-specific detail is another significant limitation of this study. Data pertaining to medication doses achieved and escalation barriers specific to individual medication classes would provide valuable insights, contributing to a greater understanding of real-world treatment benchmarks [22]. Replication in broader populations is essential to assess the generalisability and adaptability of this model beyond the private healthcare setting. Notably, patients in this study had sufficient digital access and literacy to support regular telecommunication. Future implementation programs should account for disparities in digital access and literacy, particularly among older adults, remote communities, and socioeconomically disadvantaged groups [29,63]. Special attention should also be given to equity of access and patient-centred requirements across varied population settings, noting that most patients enrolled in this study had the financial resources to access private healthcare [49]. Importantly, a 12-month follow-up analysis would strengthen the evidence base for this care model, incorporating measures such as medication adherence, cohort-wide LVEF data, and clinical outcomes, including hospitalisations and cardiovascular and all-cause mortality [4,5,6]. Nurse-led models are generally associated with reduced hospitalisations and lower healthcare costs, as demonstrated by Weinstein et al. (2022, Israel), Ledwidge et al. (2003, Ireland), a systematic review by Wu et al. (2024, USA-based economic analyses) and a meta-analysis by Ahmed et al. [25,26,48,64]. However, a detailed economic analysis of the current program would provide compelling data for policymakers and health service administrators considering broader implementation strategies [2].

## 5. Conclusions

In summary, this nurse-led, AI-supported, remote medication titration initiative improved the uptake of 4P-HF therapy and was associated with improvements in LVEF among patients with HFrEF. This model serves as a blueprint for scalable and digitally enabled interventions aimed at optimising heart failure care in real-world settings.

## Figures and Tables

**Table 1:** Patient characteristics and heart failure medication use.

Patient Data	Intervention Baseline n = 2004 ^1^	Post Intervention n = 2004	*p*-Value
Age, years	72.7 ± 11.6	-	-
Male	1444 (72.1)	-	-
Regional	732 (36.5)	-	-
Metropolitan	1271 (63.5)	-	-
LVEF, %	38.2 ± 10.8	-	-
eGFR mL/min/1.73 m^2^ (n = 1612)	61.3 ± 19.8	-	-
CKD4-5	95 (5.9)	-	-
Diabetes Mellitus (n = 1085)	187 (17.2)	-	-
**Medication class used**			
ACEI/ARB/ARNI	1779 (88.8)	1932 (96.4)	<0.001
ACEI/ARB	-	573 (28.6)	
ARNI	-	1359 (67.8)	
BB	1771 (88.4)	1944 (97.0)	<0.001
MRA	1035 (51.6)	1316 (65.7)	<0.001
SGLT2i	347 (17.3)	1481 (73.9)	<0.001
**Number of heart failure medications**			
0	55 (2.7)	3 (0.1)	<0.001
1	221 (11.0)	27 (1.3)	<0.001
2	712 (35.5)	274 (13.7)	<0.001
3	794 (39.6)	702 (35.0)	0.003
4	222 (11.1)	998 (49.8)	<0.001

Data are presented as mean ± SD, or number (percentage). ^1^ Baseline data were available for all 2004 patients unless otherwise specified. Baseline data on geography were missing for one patient. Baseline eGFR and diabetic status was not available for all patients, with eGFR data available for n = 1612 and diabetic status available for n = 1085 patients. Baseline data were not available for ACEI/ARB and ARNI separately. Regional and metropolitan geography pertains to patient location. CKD4-5 is defined as eGFR < 30 mL/min/1.73 m^2^. ACEI, angiotensin-converting enzyme inhibitors; ARB, angiotensin II receptor blockers; ARNI, angiotensin receptor blocker–neprilysin inhibitor; BB, beta blockers; CKD, chronic kidney disease; eGFR, estimated glomerular filtration rate; LVEF, left ventricular ejection fraction; MRA, mineralocorticoid receptor antagonists; SGTL2i, sodium–glucose cotransporter 2 inhibitors; TTE, transthoracic echocardiogram.

## Data Availability

The original contributions presented in this study are included in the article. Further inquiries can be directed to the corresponding author(s).

## Supplementary Materials

The following supporting information can be downloaded at https://www.mdpi.com/article/10.3390/jcm14155371/s1, Table S1: Defined target doses of study medications and a detailed description of the natural language processing tool developed for this study. Table S2: Structured ruleset for heart failure entity classification; Table S3: Software dependencies used to create the NLP model.

## Abbreviations

The following abbreviations are used in this manuscript:

4P-HF	Four-pillar heart failure
AI	Artificial intelligence
ACEI	Angiotensin converting enzyme inhibitor
ARB	Angiotensin receptor blocker
ARNI	Angiotensin receptor blocker–neprilysin inhibitor
BB	Beta blocker
CKD	Chronic kidney disease
EF	Ejection fraction
EMR	Electronic medical record
GDMT	Guideline directed medical therapy
HFrEF	Heart failure with reduced ejection fraction
HF	Heart failure
IQR	Interquartile range
LVEF	Left ventricular ejection fraction
NYHA	New York Heart Association
MRA	Mineralocorticoid receptor antagonist
NLP	Natural language processing
NT-proBNP	N-terminal prohormone of brain natriuretic peptide
RCT	Randomised control trial
SD	Standard deviation
SGLT2i	Sodium–glucose cotransporter 2 inhibitor
TTE	Transthoracic echocardiogram
