# Supplementary material for: Nurse-Led, Remote Optimisation of Guideline-Directed Medical Therapy in Patients with Heart Failure and Reduced Ejection Fraction Across Australia
Source: J Clin Med. 2025 Jul 30;14(15):5371. doi: 10.3390/jcm14155371 (PMC12347357; doi:10.3390/jcm14155371)
Supplement: Supplementary file 1 [file jcm-14-05371-s001.zip › jcm-3756966-supplementary.pdf]

Journal of Clinical Medicine

MDPI

Supplementary Material

Table S1. Defined target doses of study medications.

Medication	Maximal Target Dose
**Angiotensin converting enzyme inhibitor**	
Perindopril	10 mg daily
Ramipril	10 mg daily
Enalapril	40 mg daily
Fosinopril	40 mg daily
Captopril	150 mg daily
Quinapril	40 mg daily
Trandolapril	4 mg daily
Lisinopril	40 mg daily
**Angiotensin receptor blocker**	
Candesartan	32 mg daily
Valsartan	320 mg daily
**Angiotensin receptor neprilysin inhibitor**	
Sacubitril/Valsartan	97mg/103 mg twice daily
**Beta blocker**	
Nebivolol	10 mg daily
Bisoprolol	10 mg daily
Carvedilol (weight < 85kg)	25 mg twice daily
Carvedilol (weight > 85kg)	50 mg twice daily
Metoprolol extended release	190 mg daily
**Mineralocorticoid receptor antagonist**	
Spironolactone	25 mg daily
Eplerenone	50 mg daily
**Sodium glucose cotransporter 2 inhibitor**	
Empagliflozin	10 mg daily
Dapagliflozin	10 mg daily

Natural language processing model development

1.1. Summary

The project integrated unstructured clinical letters and structured measurements into a deterministic, rule-based NLP pipeline that extracted key entities and their values. It then applied expert-defined thresholds to stratify heart failure risk. Patient letters, discharge summaries, pathology reports, echocardiogram metrics and laboratory biomarkers were ingested, using phrase matching techniques to identify terms like "ejection fraction" and numerical expressions across documents. Further examples of extracted entities included EF 40%, N-terminal prohormone of brain natriuretic peptide (NT-proBNP) 125 pg/mL, New York Heart Association (NYHA) class III–IV. Extracted entities were fed into a rule engine containing cardiology guidelines which output a hierarchy of risk classifications.

1.2. Data Sources

All data was de-identified before processing. Structured data included echocardiogram measurements, laboratory results, blood-pressure logs and medication lists. Unstructured data included specialist letters, discharge summaries and pathology narratives.

1.3. Preprocessing

- File conversion
  - PDFs → images → text via pdf2image 1.13.1
  - RTF → plain text via striprtf 0.0.10
  - DOCX → text via docx2txt 1.0
- Tokenisation & normalisation
  - SpaCy 2.3.5 (`en_core_web_lg` NLP model) provides sentence boundaries, tokens, lemmas, and part-of-speech tags.
  - All text was lower case and non-breaking spaces were removed.
- Structured tables are ingested unchanged into Pandas dataframes.

1.4. Pattern matching engine

Phrase matching techniques were employed in the NLP tool to extract entities and values efficiently. First, pattern objects for matching were defined. Each text phrase from the dataset and template obtained from defined patterns were hashed via the shared vocabulary constructed from the text extracted from the patient dataset (letters, documents, tables etc.). All hashed patterns built a prefix tree with failure links (Aho–Corasick algortithm for efficient string search). The token ID array was scanned in a single pass linear time, despite hundreds of phrases.

1.5. Relevant code blocks

- Pattern store – `matcher_config.json` defined token patterns covering medications, cardiac measurements, symptoms and devices.
- Matcher – Patterns were compiled at start-up into spaCy's high-performance Aho–Corasick style trie; therefore every document was scanned in O(n) time regardless of pattern count.
- Negation detection – A sentence-level rule flags phrases such as "no evidence of heart failure" suppressed false positives.
- Unit & value capture – Helper regex utilities (`extract_numbers`, `extract_words`) parse numeric tokens and units (e.g., "EF = 38 %", "NT-proBNP 125 pg mL^{-1}").

1.6. Rule engine – risk and therapy assessment

After extraction, a rule engine evaluated each entity's value and context against cardiology thresholds (e.g., EF 40%, NT-proBNP 125 pg/mL, symptom keywords like "orthopnea," "paroxysmal nocturnal dyspnea," or NYHA class) to assign a "risk" classification flag. The engine's logic allowed iterative enhancement: to add or adjust rules without retraining, enabling rapid response to new guidelines or observed edge cases. The `Heart_failure.py` aggregated extractions from clinical documents relevant to the patient and executed a transparent ruleset that mirrored consensus guideline examples (Table B). Because logic was codified as Python statements, new rules and drug classes could be added without retraining.

Table S2: Structured ruleset for heart failure entity classification

**Criteria**	**Rule example**	**Outcome**
Systolic function	Lowest LVEF < 40 %	Flag "definite HFrEF"
Biomarkers	NT-proBNP > 450 pg mL^{-1} (< 50 years)	Elevates risk tier
Structure	LA volume > 34 mL m^{-2} or LVMI > 115 g m^{-2}	Supports Heart Failure with Preserved Ejection Fraction diagnosis

Symptoms	≥ 2 HF keywords or explicit NYHA class	Validates clinical syndrome
Medication gap	< 50 % of ACEI target dose	"Under-treated" tag

1.7. *Sensitivity and specificity of the NLP*

The NLP extraction algorithm was evaluated for accuracy in identifying HFrEF diagnoses. 100 random patients were sampled from the Advara dataset, 11 of whom had HFrEF diagnosed by a cardiologist. The NLP identified 10 true positives, 0 false positives, 89 true negatives and 1 false negative. Therefore the sensitivity of the NLP in diagnosing HFrEF was 90.9% and the specificity was 100%.

1.8. *Software dependencies*

Table S3: Software dependencies used to create the NLP model

**Package**	**Version**	**Function**
pdf2image	1.13.1	PDF page rasterisation
striprtf	0.0.10	RTF → text
joblib	1.4.2	Parallel utilities
nltk	3.8.1	Ancillary text parsing
docx2txt	1.0	DOCX → text
spacy	2.3.5	NLP core
python-docx	0.8.10	Report generation
dirtyjson	1.0.5	Lenient JSON loader
pandas	1.5.3	Dataframes & I/O
numpy	1.23.5	Numeric ops

Disclaimer/Publisher's Note: The statements, opinions and data contained in all publications are solely those of the individual author(s) and contributor(s) and not of MDPI and/or the editor(s). MDPI and/or the editor(s) disclaim responsibility for any injury to people or property resulting from any ideas, methods, instructions or products referred to in the content.
